# 1451. *Elizabethkingia spp*. Outbreak in a Ventilator-capable Skilled Nursing Facility, Chicago 2023

**DOI:** 10.1093/ofid/ofad500.1288

**Published:** 2023-11-27

**Authors:** Sidney Thigpen, Kelly Walblay, Hira Adil, Shane Zelencik, Christy Zelinski, Kathryn Nelson, Brian Cox, John R McQuiston, Janice Turner, Karrie-Ann Toews, Do Young Kim, Stephanie R Black

**Affiliations:** Chicago Department of Public Health, Chicago, Illinois; Chicago Department of Public Health, Chicago, Illinois; CDPH, Chicago, Illinois; Chicago Department of Public Health, Chicago, Illinois; Chicago Department of Public Health, Chicago, Illinois; Illinois Department of Public Health, West Chicago, Illinois; Illinois Department of Public Health, West Chicago, Illinois; Centers for Disease Control and Prevention, Atlanta, Georgia; Chicago Department of Public Health, Chicago, Illinois; Centers for Disease Control and Prevention, Atlanta, Georgia; Chicago Department of Public Health, Chicago, Illinois; Chicago Department of Public Health, Chicago, Illinois

## Abstract

**Background:**

In July 2022, the Chicago Department of Public Health (CDPH) was notified of four residents with *Elizabethkingia (Ek)* infections at a ventilator-capable skilled nursing facility (vSNF A). The facility reviewed microbiology records, identified six additional cases and CDPH initiated an investigation.

**Methods:**

A case-patient was defined as a resident at vSNF A with *Ek spp.* identified from a clinical specimen. Information was collected via case report form regarding risk factors for *Ek* infection. Site visits were conducted to observe dialysis, wound care practices, and water disruption. Environmental and clinical isolates were sequenced by the CDC to assess genetic relatedness.

**Results:**

January 2021-2023, 14 case-patients were identified with *Ek* infection: 11 bacteremias, two pneumonias, and one had both. Two case-patients died. Nine case-patients were hemodialyzed, six required ventilator support, and all case-patients had decubitus ulcers. Seven had exposures to tap-water during care. Site visits revealed potential exposure through bathing or healthcare worker hands contaminated by tap-water, personal items on sink-tops, and sterile supplies stored by sinks. Water quality parameters verified chlorine levels above minimum standards by the Illinois Pollution Control Board. Among environmental samples collected, *E. anophelis* was isolated from first-catch water in the room of a ventilated case-patient. Whole genome sequencing indicated this isolate was closely related to the clinical isolate of the case-patient in the room and a case-patient on a different floor. Both patients were hemodialyzed and exposed to tap-water for bathing.

**Conclusion:**

Infection control recommendations included avoiding tap-water for bathing, not disposing body-fluid in sinks, routine flushing, disinfecting or removing aerators, and disinfecting sink drains with scheduled foaming disinfectant application. This investigation demonstrates a persistent outbreak of *Ek* in a vSNF population, likely related to contaminated tap-water. We postulate that *Elizabethkingia* persisted in water system biofilms, despite measurable chlorine levels, leading to patient exposure by contaminated healthcare worker hands or through tap water bathing in the presence of decubitus ulcers.

**Disclosures:**

**All Authors**: No reported disclosures

